# Blue Light Exposure Induces Changes Associated With Senescence and Skin Aging in Human Dermal Fibroblasts

**DOI:** 10.1096/fba.2026-00200

**Published:** 2026-09-22

**Authors:** Helen McNish, Mruthyunjaya Swamy Mathapathi, Katarzyna Figlak, Anita Damodaran, Mark Birch‐Machin

**Affiliations:** ^1^ Dermatological Sciences, Translational and Clinical Research Institute Newcastle University London UK; ^2^ Unilever R&D Bangalore India; ^3^ Unilever Bioscience Innovation Hub, King's College London London UK

**Keywords:** aging, blue light, senescence, skin

## Abstract

Blue light is becoming increasingly prevalent in our daily lives. However, the possible deleterious effects of blue light exposure on the skin have been largely overlooked. We have previously shown that blue light has the ability to induce mitochondrial DNA (mtDNA) damage and dysfunction in human dermal fibroblasts (HDFn). However, whether blue light is able to induce senescence in skin has yet to be investigated. Cellular senescence is a key part of the aging process and is heavily associated with an aging phenotype. Our study involved exposing HDFn to varying doses of blue light before assessing the expression of known senescence markers using both immunofluorescence and gene expression qPCR. We also assessed beta‐galactosidase activity using a senescence‐associated beta‐galactosidase (SA β‐gal) staining kit. Blue light induces increased γH2AX expression (*p* = ≤ 0.0001), indicating its ability to induce DNA double‐strand breaks. P21, a known marker of senescence, is increased in response to blue light, as seen in both immunofluorescence and gene expression, where a 15‐fold increase is reported in cells exposed to 50 J/cm^2^ blue light (*p* = ≤ 0.001). Importantly, NFκB also displayed a dose‐dependent increase in expression following blue light exposure, with 25 J/cm^2^ (*p* = ≤ 0.0001) and 50 J/cm^2^ (*p* = ≤ 0.0001) inducing maximal nuclear translocation. SA β‐gal activity also increased in response to blue light, with 25 J/cm^2^ being the minimum dose required to induce statistical significance (*p* = ≤ 0.01) when compared to a non‐irradiated control group. Taken together, we demonstrate that blue light may have the ability to induce not only mtDNA damage but also nuclear DNA (nDNA) damage. This, combined with the increased expression of senescent markers and SA‐β‐gal activity, suggests that blue light could induce cellular senescence while also eliciting the bystander effect in neighboring cells due to increased NFκB nuclear translocation and associated reactive oxygen species (ROS) production. These findings emphasize the need for further research into the potential deleterious effects of senescence in skin and for future interventions to attenuate these effects.

## Graphical abstract

We are being exposed to increasing amounts of blue light on a daily basis, both from the sun and electronic devices. Our research builds on previous novel work and suggests that blue light is capable of inducing not only mitochondrial but also nuclear DNA damage in human dermal fibroblasts. It is also shown to increase expression of known senescence markers and, importantly, senescence‐associated beta‐galactosidase. Taken together, we demonstrate that blue light may be able to induce senescence in human dermal fibroblasts, which may contribute to premature aging of the skin.

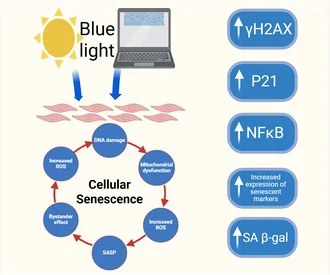

**Abbreviations**
ATP: adenosine triphosphate
BCL2: BCL2 apoptosis regulator
cDNA: complementary DNA
DAPI: 4′,6‐diamidino‐2‐phenylindole
DMEM: Dulbecco's modified eagle medium
DNA: deoxyribonucleic acid
ECM: extracellular matrix
GAPDH: glyceraldehyde 3‐phosphate dehydrogenase
HDFn: neonatal human dermal fibroblasts
LEDs: light‐emitting diodes
MMP: matrix metalloproteinase
mtDNA: mitochondrial DNA
NFκB: nuclear factor kappa B
OPN3: opsin 3
P16: cyclin‐dependent kinase inhibitor 2A
P21: cyclin‐dependent kinase inhibitor 1A
P53: tumor protein p53
PBS: phosphate‐buffered saline
qPCR: quantitative polymerase chain reaction
RNA: ribonucleic acid
ROS: reactive oxygen species
SASP: senescence‐associated secretory phenotype
UV: ultraviolet
UVR: ultraviolet radiation
γH2AX: gamma‐H2AX

## 1 Introduction

Modern society exposes us to increasing levels of blue light, with sources ranging from sunlight, electronic devices, and light‐emitting diodes (LEDs) [1]. The 50 J/cm^2^ of blue light is equivalent to 1 h of solar exposure expected at noon in midsummer in the Mediterranean at 45**°** latitude [2]. Blue light is closest in terms of wavelength to ultraviolet (UV), with a wavelength between 400 to 500 nm; previous research has shown that blue light is capable of inducing effects on skin similar to those seen in ultraviolet radiation (UVR) [1, 3]. Recent research links blue light with decreased cell viability and reduced proliferation [4, 5]. This is combined with altered cellular morphology in both keratinocytes and fibroblasts [5, 6]. This suggests that blue light has the ability to penetrate both the epidermis and the dermis, with fibroblasts developing a rounded appearance and keratinocytes showing early differentiation [5, 6, 7]. The ability of blue light to penetrate the dermis is confirmed by increased matrix metalloproteinase (MMP) expression, which is linked to collagen degradation and reduced skin integrity [8, 9]. Blue light also causes hyperpigmentation, with both immediate and persistent pigment darkening [3, 8, 9, 10]. Changes in pigmentation have been associated with opsin 3 (OPN3) activation [11]. The immune response can be altered, with both innate and adaptive immunity affected, causing increased inflammation and decreased immune response; this is potentially linked to increased T cell apoptosis. Recently, blue light has been shown to induce mitochondrial damage and dysfunction in human dermal fibroblasts [12]. Blue light induces mitochondrial DNA (mtDNA) strand breaks and mitochondrial dysfunction, which culminates in decreased adenosine triphosphate (ATP) production. Oxidative stress has also been linked to increased reactive oxygen species (ROS) production in response to blue light [12, 13]. Similar to visible light, this increase in ROS could be attributed to carotenoid depletion, which has also been reported in skin exposed to blue light [4, 14]. Premature skin aging was previously associated with only UVR; however, research now demonstrates that blue light exposure is also associated with premature skin aging [9, 13, 15]. This is known as extrinsic skin aging and is characterized by deep wrinkles, leathery appearance, dry skin, and uneven pigmentation [16]. These findings raise the possibility of future research into sunscreen development that may protect us from the effects of blue light [9, 15].

Cellular senescence is defined as a loss of deoxyribonucleic acid (DNA) replication and lack of proliferation due to premature cell cycle arrest [17]. It is a defense mechanism against tumorigenesis and uncontrolled cell division; however, it is also associated with aging [17]. There are two types of senescence. Firstly, telomere‐dependent senescence, which is a natural consequence of aging due to DNA replication, cell division, and subsequent telomere shortening. Secondly, telomere‐independent senescence, which is stress‐induced via DNA damage and the DNA damage response mechanisms [18]. Mitochondrial damage has also been associated with cellular senescence [18]. Senescent cells are non‐functional but also have increased expression of BCL2 apoptosis regulator (BCL2), a pro‐survival protein that prevents apoptosis of senescent cells [19]. These senescent cells are also known to express a senescence‐associated secretory phenotype (SASP), which is a cocktail of pro‐inflammatory cytokines and ROS mediators capable of inducing senescence in neighboring cells; this is known as the bystander effect [19]. The bystander effect is associated with an increase in reactive oxygen species and increased nuclear factor kappa B (NFκB) nuclear translocation, which then induces DNA damage responses in neighboring cells [18]. Multiple studies have established a correlation between the accumulation of senescent cells and an aging skin phenotype [20].

Previous research has established a correlation between increased cellular senescence, expression of β‐galactosidase, a known senescence marker, and aging in skin [21, 22]. The ability of DNA damage to induce senescence opens up the possibility that UVR, one of the main mechanisms of DNA damage, may have the ability to induce senescence in skin [23]. This makes research into the effects of blue light warranted. The DNA damage response may also induce the activation of cyclin‐dependent kinase inhibitor 1A and 2A, known as P21 and P16, respectively, leading to cellular senescence [22]. P21 is associated with early‐onset senescence, with P16 upregulation occurring at a later stage [24]. Senescence in fibroblasts can be observed by altered cell morphology, such as cellular enlargement with a flattened appearance [24]. Senescence is known to impair skin physiology, leading to many phenotypes associated with aging [25]. Some of these phenotypes include loss of skin elasticity and integrity, reduced wound healing, increased inflammation, and reduced barrier to insults [26]; however, research exploring the ability of blue light to induce cellular senescence in skin has yet, to our knowledge, to be conducted. This study investigates whether blue light has the ability to induce senescence in human dermal fibroblasts, subsequently leading to premature aging of the skin.

## 2 Materials and Methods

### 2.1 HDFn Cell Culture

Neonatal Human dermal Fibroblasts (HDFn) (Invitrogen, UK) were cultured in T175 flasks (Sarstedt, Germany) with Dulbecco's Modified Eagle Medium (DMEM) containing 4.5 g/L glucose, L‐glutamine, sodium pyruvate, and sodium bicarbonate (Gibco, UK), supplemented with 10% Foetal Bovine Serum (FBS) (Merck, Germany) and 1% Penicillin–Streptomycin (Pen/Strep) (SLS, UK). This will be described as complete DMEM for the duration of the study. Cells were maintained in a humidified incubator, with a temperature of 37°C and 5% CO_2_. All biological repeats were performed in triplicate (*n* = 3).

### 2.2 Blue Light Irradiation

Cells were irradiated with blue light ranging from 400 to 500 nm using a Newport solar simulator (Newport Oriel model 91,292, 1,000 W, MKS Instruments Inc., USA), with a beam spot area of 4 × 4 in. To isolate blue light only for the solar simulator, filters were used. Inside the solar simulator housing, a 400 nm longpass (UQG Optics, UK), 510 nm shortpass (Asahi, Japan), and infrared (IR) (Asahi, Japan) filters were placed in a holder. The blue light output from the solar simulator was 10 mW/cm^2^, as measured using a FLAME‐S‐XR1‐ES spectroradiometer fitted with a CC‐3‐DA direct‐attach cosine corrector with a Spectralon diffuser (OceanOptics, Netherlands). Throughout the study, blue light doses ranged from 5 to 100 J/cm^2^, with an irradiation duration of 8 min 17 s to achieve 5 J/cm^2^. A non‐irradiated, foil‐protected group was designated as the control. All doses and irradiation durations were calculated using the following equations:

$$\text{Irradiation time}\;\left(\text{secs}\right)=\left(\frac{\text{Dose}\;\left(J/{\mathrm{cm}}^{2}\right)}{\text{Irradiance}\;\left(\mathrm{mW}/{\mathrm{cm}}^{2}\right)}\;\right)\times 1000$$

$$\text{Dose}\;\left(J/{\mathrm{cm}}^{2}\right)=\frac{\left(\text{Irradiance}\;\left(\mathrm{mW}/{\mathrm{cm}}^{2}\right)\times \text{Time}\left(\text{secs}\right)\right)}{1000}$$

### 2.3 Gene Expression Assay

HDFn cells were seeded at a density of 150,000 cells per 35 mm dish in 2 mL complete DMEM. Following blue light irradiation at the desired doses, all cells were incubated at 37°C with 5% CO_2_ for 16 h. A non‐irradiated, foil‐protected group was designated as the control. HDFn cell pellet isolation was performed by aspirating complete DMEM, washing all cells with 2 mL PBS, and then adding 500 μL Trypsin–EDTA, which covered the surface of each 35 mm dish. 1 mL complete DMEM was subsequently added for trypsin neutralization. A total suspension volume of 1.5 mL from each dish was placed in separate 1.5 mL Eppendorf tubes (Eppendorf, Germany). All tubes were then centrifuged at 500 × *g* for 5 min. The supernatant was aspirated, and the pellet was washed and resuspended in 1.5 mL PBS before being centrifuged again at 500 *x g* for 5 min. The supernatant was once again aspirated without disturbing the pellet, before proceeding immediately to ribonucleic acid (RNA) extraction. Total RNA from each irradiation dose was isolated using the RNeasy mini kit (Qiagen, UK), following the manufacturer's protocol. RNA concentrations were then measured using a NanoDrop 2000 (NanoDrop Technologies LLC, Thermo Fisher Scientific, USA). RNA purity was assessed using the 260/280 nm ratio. Reverse transcription was performed using the High Capacity cDNA Reverse Transcription Kit (Thermo Fisher Scientific, USA), following the manufacturer's step‐by‐step protocol with RNase inhibitor (https://www.thermofisher.com/order/catalog/product/4368814?SID=srch‐srp‐4368814. Thermo Fisher Scientific, USA). The 10 μL of 2X reverse transcription master mix was prepared according to the manufacturer's guidelines and placed into each reaction tube. Following that, 10 μL of RNA sample was also added to each reaction tube, providing a final reaction volume of 20 μL. Prior to each quantitative Polymerase Chain Reaction (qPCR) reaction, cDNA was diluted with DNase‐free water to a concentration of 20 ng/μL. TaqMan gene expression master mix was added to each designated well of a 96‐well polymerase chain reaction (PCR) reaction plate (MicroAmp EnduraPlate Optical 96‐Well Clear Reaction Plate, Thermo Fisher Scientific, USA), followed by cDNA, to give a final reaction volume of 20 μL. All TaqMan primers were supplied by Thermo Fisher Scientific, USA, with 100% efficiency confirmed (https://documents.thermofisher.com/TFS‐Assets/LSG/Application‐Notes/cms_040377.pdf). To normalize the data, glyceraldehyde 3‐phosphate dehydrogenase (GAPDH) was used as a housekeeping gene. Relative gene expression was calculated using the 2^−ΔΔCt^ method.

### 2.4 Immunocytochemistry

HDFn cells were seeded at a density of 150,000 cells per 35 mm dish in 2 mL complete DMEM. Following blue light irradiation at the desired doses with a Newport solar simulator with appropriate filters, and incubation for the desired time, cells were washed three times with PBS before fixing with 4% paraformaldehyde for 10 min. Upon completion of fixation, cells were permeabilized with 0.3% Triton‐X‐100. Following washing with PBS, all cells were blocked with 10% normal goat serum in PBS. Cells were then incubated overnight at 4֯C with primary antibody for γH2AX (1/1000, Cell Signaling Technology, RRID: AB_2118010), P21 (1/1000, Abcam, RRID: AB_10860537), or NFκB (1/1000, Cell Signaling Technology, RRID: AB_10859369) in antibody incubation buffer (1% bovine serum albumin in PBS). Cells were then washed with PBS before application of the secondary antibody Alexa Fluor 488 for 1 h, protected from light. Upon removal of the secondary antibody, cells were washed again with PBS before cell nuclei counterstaining with ProLong Antifade Diamond Mountant with DAPI. Cells were visualized using a Zeiss LSM880 NLO Multiphoton microscope (Zeiss, Germany). All image quantification was completed using ImageJ software (NIH, USA; RRID: SCR_003070). For analysis of γH2AX nuclear foci, P21 nuclear intensity, and NFκB nuclear translocation, quantitative analysis of mean nuclear intensity per pixel was performed as a measure of total protein in the region of interest.

### 2.5 Senescence‐Associated Beta‐Galactosidase Staining

HDFn cells were seeded at a density of 150,000 cells per 35 mm dish in 2 mL complete DMEM. Following irradiation at the desired doses of blue light, cells were stained using the Senescence Cells Histochemical Staining Kit (Sigma‐Aldrich, UK). After complete PBS removal, cells were fixed using 1.5 mL per dish of the provided fixing solution (20% formaldehyde, 2% glutaraldehyde, 70.4 mM Na_2_ HPO_4_, 14.7 mM KH_2_PO_4_, 1.37 M NaCl and 26.8 mM KCl), before washing with PBS. Immediately after removal of the final PBS wash, cells were stained using freshly prepared staining solution (400 mM Potassium Ferricyanide, 400 mM Ferrocyanide, 40 mg/mL X‐gal solution) and incubated for 16 h at 37°C with no CO_2_ to prevent pH changes and maintain optimal staining conditions. For visualization, the staining solution was replaced with PBS, and cells were imaged under an EVOS XL Core Cell Imaging System microscope. Image quantification was conducted using ImageJ software (NIH, USA). For each desired image, color deconvolution was performed, and the image was thresholded. Following thresholding, which created a binary image, the area designated as positive for SA β‐gal was expressed as an overall percentage area of the image.

### 2.6 Statistical Analysis

To enable statistical analysis to be conducted, all biological repeats were performed in triplicate (*n* = 3). Due to the nature of the study, a formal power calculation was not required, as it is a cell‐based in vitro study as opposed to a double blind randomized in vivo study. To assess whether data were normally distributed, a D'Agostino‐Pearson omnibus test was used. One‐way ANOVA corrected for multiple comparisons using Tukey's test, or Kruskal–Wallis corrected for multiple comparisons using Dunn's test, was conducted based on normality test results (see figure legends). All statistical analyses were conducted in GraphPad Prism, version 10.6.1(RRID: SCR_002798).

## 3 Results

### 3.1 Blue Light at Low and Higher Doses Induces Increased γH2AX Expression

**FIGURE 1:**
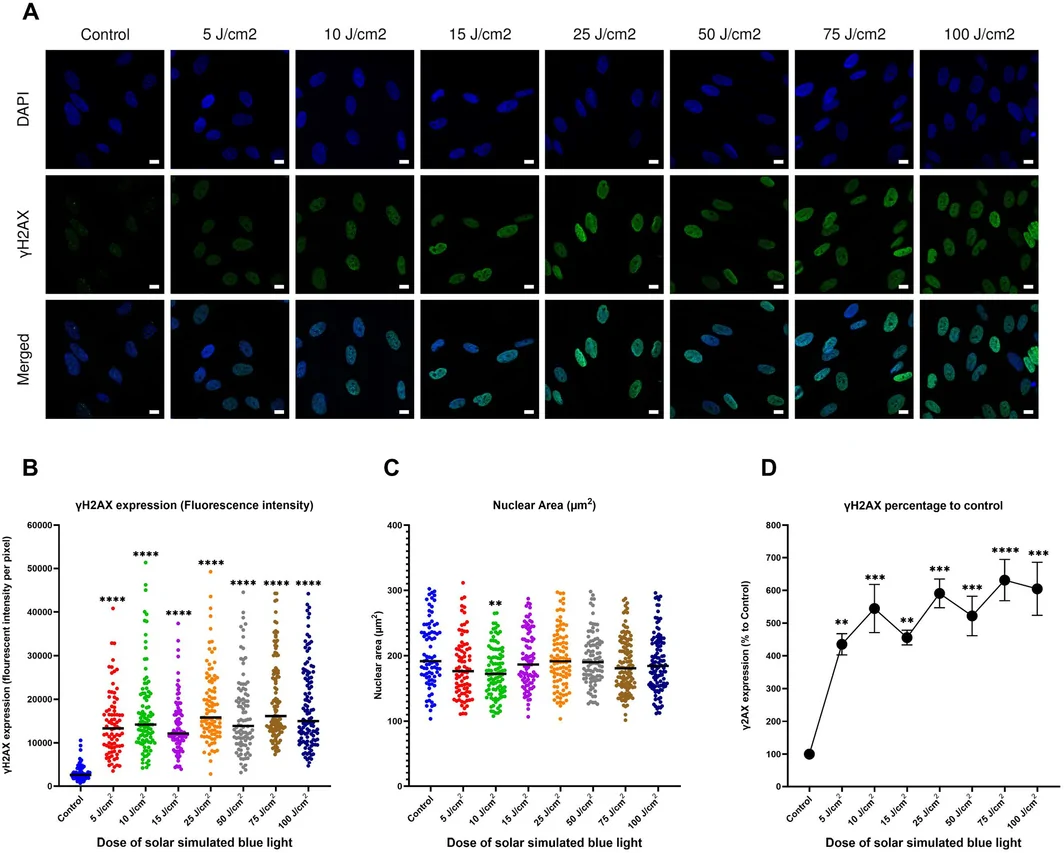
Analysis of the effect of blue light on DNA double‐strand breaks in HDFn with γH2AX immunofluorescence. HDFn were exposed to blue light doses ranging from 5 to 100 J/cm^2^. Cells were fixed immediately after irradiation, stained with an anti‐γH2AX antibody, and the nuclei counterstained with 4, 6‐diamidino‐2‐phenylindole (DAPI). Visualization was conducted using fluorescence microscopy, and quantitative analysis was completed using ImageJ. (A) Representative images of HDFn exposed to blue light and stained with an anti‐γH2AX antibody. (B) γH2AX expression quantified using mean fluorescent intensity per pixel. (C) Nuclear area (μm) of each quantified cell. (D) γH2AX expression expressed as a percentage relative to the non‐irradiated control. Scale bars on images represent 10 μm. *n* = 3. D'Agostino‐Pearson omnibus test was used to assess normality of the data. For fluorescence intensity and nuclear area, the Kruskal–Wallis test, corrected for multiple comparisons using Dunn's test, was used. For percentage change, a one‐way ANOVA with Tukey's multiple comparisons test was performed to assess differences between doses and the control group. **p ≤ 0.01, ***p ≤ 0.001, ****p ≤ 0.0001. Data represent the median for fluorescence intensity and nuclear area. Data represent the mean ± SEM for γH2AX percentage relative to the control.

Given our previous research detailing the ability of blue light to induce mtDNA damage, we subsequently investigated its ability to cause DNA damage by way of double‐strand breaks using γH2AX immunofluorescence. Dermal fibroblasts were irradiated with doses of blue light ranging from 5 to100 J/cm^2^, before immediate fixation and proceeding with immunofluorescence. A non‐irradiated, foil‐protected group was designated as the control. γH2AX expression was increased at all doses, with visibly increased expression across all images (Figure 1A). When quantified by the mean nuclear intensity per pixel of each cell, statistical significance was displayed at all doses (*p* = ≤ 0.0001) (Figure 1B). Quantified as a percentage, a 400%–500% increase in γH2AX expression was observed in cells exposed to 5 J/cm^2^ (*p* = ≤ 0.01), 10 J/cm^2^ (*p* = ≤ 0.0001), and 15 J/cm^2^ (*p* = ≤ 0.01) (Figure 1D). γH2AX expression continued to increase in cells exposed to higher doses of blue light, peaking at 600% increased expression in cells exposed to 75 J/cm^2^ (p = ≤ 0.0001) and 100 J/cm^2^, respectively (p = ≤ 0.001) (Figure 1D). Nuclear area of all cells displayed little change in response to blue light, with only cells exposed to 10 J/cm^2^ displaying a statistically significant decrease in nuclear area (*p* = ≤ 0.01).

### 3.2 P21, An Early Senescence Marker, Is Upregulated in Response to Blue Light

**FIGURE 2:**
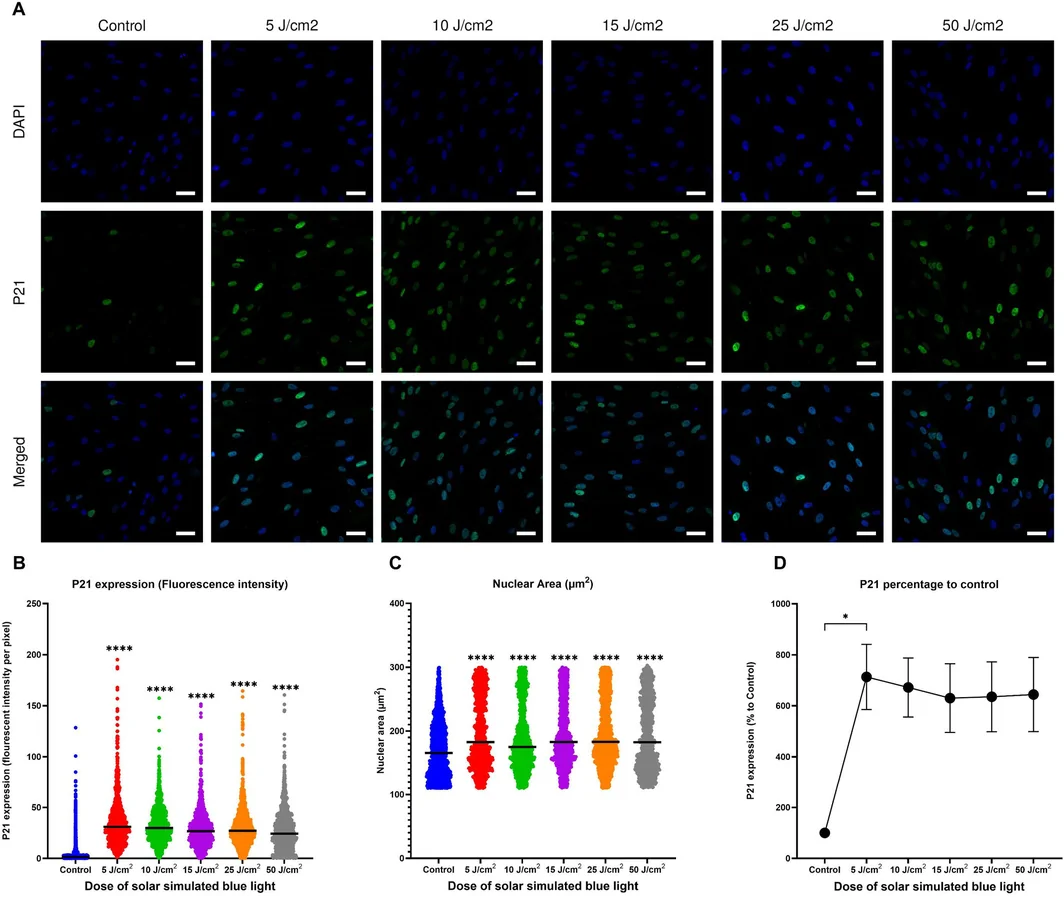
Analysis of the effect of blue light on P21 expression using immunofluorescence. HDFn were exposed to blue light doses ranging from 5 to 50 J/cm^2^. Cells were fixed 16 h after irradiation, stained with anti‐P21 antibody, and the nuclei counterstained with 4, 6‐diamidino‐2‐phenylindole (DAPI). Visualization was conducted using fluorescence microscopy. (A) Representative images of HDFn exposed to blue light and stained for anti‐P21 antibody. (B) P21 expression was quantified using mean fluorescent intensity per pixel. (C) Nuclear area (μm) of each quantified cell (C) P21‐positive cells expressed as a percentage relative to non‐irradiated control. (D) P21‐positive cells expressed as a fold change relative to non‐irradiated control. Scale bars on images represent 50 μm. *n* = 3. D'Agostino‐Pearson omnibus test was used to assess normality of the data. For fluorescence intensity and nuclear area, the Kruskal–Wallis test, corrected for multiple comparisons using Dunn's test, was used. For percentage change, a one‐way ANOVA with Tukey's multiple comparisons test was performed to assess differences between doses and the control group. *p ≤ 0.05, **p ≤ 0.01, ***p ≤ 0.001, ****p ≤ 0.0001. Data represent the median for fluorescence intensity and nuclear area. Data represent the mean ± SEM for P21 percentage relative to control.

To assess the impact of blue light on P21 expression, which is strongly associated with early‐stage senescence, immunofluorescence was conducted. Dermal fibroblasts were irradiated with doses of blue light ranging from 5 to 50 J/cm^2^, before fixation 16 h after irradiation and subsequent immunofluorescence. A non‐irradiated, foil‐protected group was designated as the control. Once again, P21 expression was increased at all doses of blue light, as seen in all images (Figure 2A). When quantified by the mean nuclear intensity per pixel of each cell, statistical significance was displayed once again at all doses (*p* = ≤ 0.0001) (Figure 2B). Nuclear cell area was elevated consistently across all doses, with statistical significance observed at all doses when compared to the non‐irradiated, foil‐protected control (*p* = ≤ 0.0001) (Figure 2C). When P21 expression was quantified as a percentage of the control, statistical significance was displayed at 5 J/cm^2^, where a 700% increase in P21 expression was observed (Figure 2D). P21 expression was notably upregulated in all doses, with greater than a 600% increase in expression reported throughout; however, statistical significance was not reached (Figure 2D).

### 3.3 Blue Light Induces Increased Nuclear Translocation of NFκB, a Key Mediator of the Senescent Bystander Effect

**FIGURE 3:**
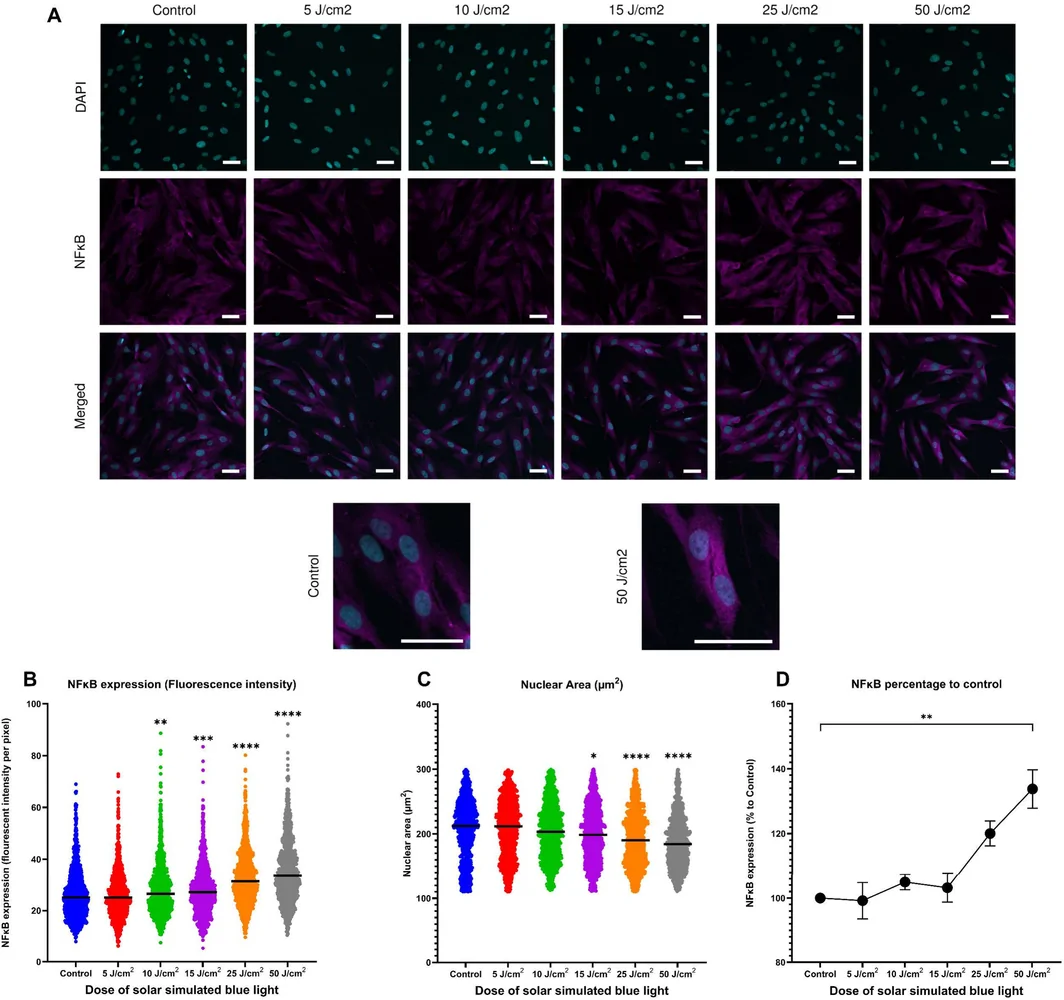
Analysis of the effect of blue light on NFκB expression using immunofluorescence. HDFn were exposed to blue light doses ranging from 5 to 50 J/cm^2^. Cells were fixed 6 h after irradiation, stained with an anti‐NFκB antibody, and the nuclei counterstained with 4, 6‐diamidino‐2‐phenylindole (DAPI). Visualization was conducted using fluorescence microscopy; quantitative analysis was completed using ImageJ. (A) Representative images of HDFn exposed to blue light and stained with an anti‐NFκB antibody. (B) NFκB expression was quantified using mean fluorescent intensity per pixel. (C) Nuclear area (μm) of each quantified cell. (D) NFκB expression expressed as a percentage relative to the non‐irradiated control. Scale bars on images represent 50 μm. *n* = 3. D'Agostino‐Pearson omnibus test was used to assess normality of the data. For fluorescence intensity and nuclear area, the Kruskal–Wallis test, corrected for multiple comparisons using Dunn's test, was used. For percentage change, a one‐way ANOVA with Tukey's multiple comparisons test was performed to assess differences between doses and the control group. *p ≤ 0.05, **p ≤ 0.01, ***p ≤ 0.001, ****p ≤ 0.0001. Data represent the median for fluorescence intensity and nuclear area. Data represent the mean ± SEM for NFκB percentage relative to control.

Following the induction of P21, associated with early senescence, it was important to establish whether the senescent bystander effect may be induced. This was investigated with NFκB immunofluorescence and assessment of nuclear translocation. Dermal fibroblasts were irradiated with doses of blue light ranging from 5 to 50 J/cm^2^, before fixation 6 h after irradiation and subsequent immunofluorescence. A non‐irradiated, foil‐protected group was designated as the control. NFκB displayed a dose‐dependent increase in nuclear translocation, as seen throughout the images (Figure 3A). This was confirmed by quantification of NFκB in each nucleus using fluorescent intensity per pixel. Statistically significant increases were observed in the groups exposed to 10 J/cm^2^ (*p* = ≤ 0.01), 15 J/cm^2^ (*p* = ≤ 0.001), 25 J/cm^2^ (*p* = ≤ 0.0001), and 50 J/cm^2^ (*p* = ≤ 0.0001) of blue light (Figure 3B). When expressed as a percentage compared to the non‐irradiated control, NFκB once again displayed a dose‐dependent response, with a 120% increase in cells exposed to 25 J/cm^2^ blue light (Figure 3D). This increased to a statistically significant 135% increase in cells exposed to 50 J/cm^2^ blue light (*p* = ≤ 0.01) (Figure 3D). Nuclear area displayed a dose‐dependent decrease in response to blue light. In particular, this can be seen in groups exposed to 15 J/cm^2^ (*p* = ≤ 0.05), 25 J/cm^2^ (*p* = ≤ 0.0001), and 50 J/cm^2^ (*p* = ≤ 0.0001) blue light (Figure 3C).

### 3.4 Blue Light Alters the Expression of Genes Associated With Senescence

**FIGURE 4:**
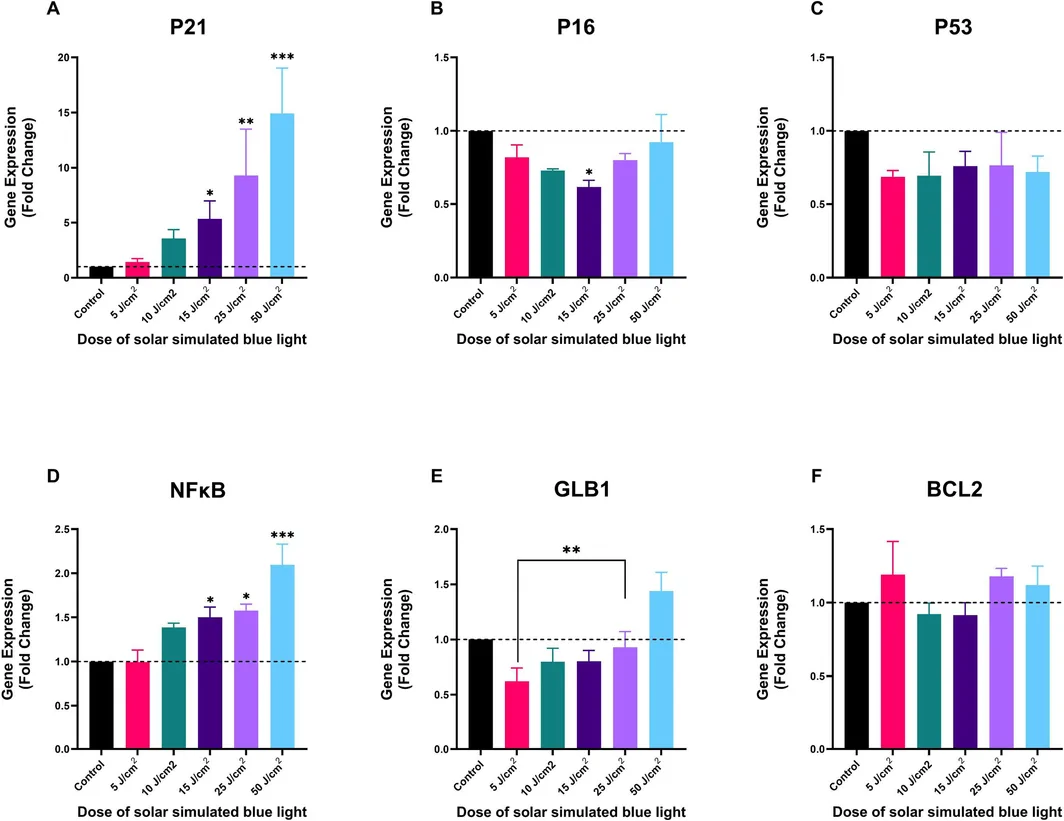
Gene expression of senescent biomarkers following blue light irradiation. HDFn cells were cultured and seeded into 35 mm tissue culture dishes. Cell seeding density was 150,000 cells in 2 mL complete DMEM. Following irradiation with blue light at the designated doses (15–50 J/cm^2^), cells were cultured for 16 h after irradiation before RNA extraction was performed and cDNA was synthesized. TaqMan gene expression qPCR was performed to assess changes in gene expression. A non‐irradiated, foil‐protected group was designated as the control and is represented as a horizontal dotted line at 1.0 on the y‐axis. A one‐way ANOVA with Tukey's multiple comparisons test was performed to assess differences between doses and irradiation time points. *p ≤ 0.05, **p ≤ 0.01, ***p ≤ 0.001, ****p ≤ 0.0001. Data represent mean ± SEM. *n* = 3.

Following clear visualization and quantification of increased expression in γH2AX, P21, and NFκB, which are all associated with the onset of senescence, it was essential to investigate the gene expression of senescence markers. Dermal fibroblasts were irradiated with doses of blue light ranging from 5 to 50 J/cm^2^, before RNA extraction was performed 16 h after irradiation. Following synthesis of cDNA, gene expression was assessed by quantitative real‐time polymerase chain reaction (qPCR). A non‐irradiated, foil‐protected group was designated as the control. P21 displayed a marked increase in gene expression (Figure 4A), which is supported by our immunofluorescence findings. Notable increased expression was observed in the group exposed to 15 J/cm^2^ (*p* = ≤ 0.05) of blue light. P21 expression continued to increase dose‐dependently, with a 9‐fold increase in cells exposed to 25 J/cm^2^ (*p* = ≤ 0.01), and a 15‐fold increase in the group exposed to 50 J/cm^2^ (*p* = ≤ 0.001) compared to the non‐irradiated control (Figure 4A). In contrast, P16 displayed consistent downregulation compared to the non‐irradiated control. Statistical significance was observed in the group exposed to 15 J/cm^2^ blue light (*p* = ≤ 0.05) (Figure 4B). NFκB displayed a dose‐dependent increase in gene expression (Figure 4D), which is also supported by immunofluorescence results. A 1.5‐fold increase in NFκB expression was observed in cells exposed to 15 J/cm^2^ blue light (*p* = ≤ 0.05), which increased to 1.6‐fold in the 25 J/cm^2^ group (*p* = ≤ 0.05). Ultimately, expression increased 2‐fold when cells were exposed to 50 J/cm^2^ (*p* = ≤ 0.001), confirming a dose‐dependent response (Figure 4D). GLB1, which encodes senescence‐associated beta‐galactosidase (SA β‐gal), displayed mixed expression following blue light exposure (Figure 4E). GLB1 was downregulated in the group exposed to 5 J/cm^2^ (*p* = ≤ 0.01). GLB1 expression displayed an upward trend in the group exposed to 50 J/cm^2^ blue light; however, statistical significance was not reached (Figure 4E). P53, strongly associated with cell cycle regulation, exhibited consistent downregulation at all doses, without statistical significance reported (Figure 4C). BCL2, a pro‐survival protein often seen in senescent cells, also displayed mixed expression in response to blue light irradiation (Figure 4F). However, a trend towards increased expression, without statistical significance, can be observed in cells exposed to both 25 J/cm^2^ and 50 J/cm^2^ (Figure 4F).

### 3.5 Senescence‐Associated Beta‐Galactosidase Increases Upon Exposure to Blue Light

**FIGURE 5:**
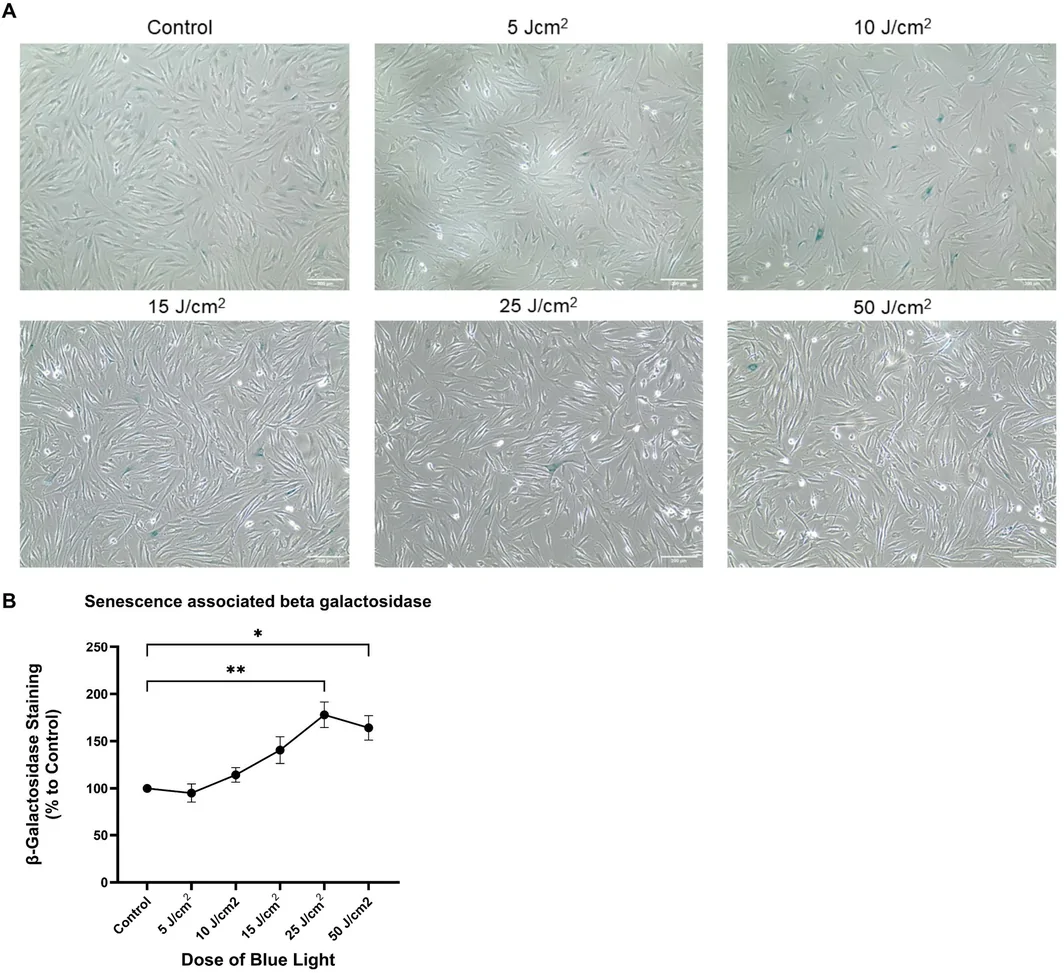
Analysis of senescence‐associated beta‐galactosidase staining in human dermal fibroblasts after blue light irradiation. Cells were irradiated with 5–50 J/cm^2^ of blue light before fixing, blocking, and staining for senescence‐associated beta‐galactosidase 16 h after blue light exposure. Quantitative analysis was performed using ImageJ. (A) Representative images of HDFn following senescence‐associated beta‐galactosidase staining using brightfield microscopy. Scale bars represent 200 μm. (B) Senescence‐associated beta‐galactosidase expression expressed as a percentage relative to the non‐irradiated control. A one‐way ANOVA with Tukey's multiple comparisons test was performed to assess differences between doses and irradiation time points. *p ≤ 0.05, **p ≤ 0.01, ***p ≤ 0.001, ****p ≤ 0.0001. Data represent the median for fluorescence intensity and nuclear area. Data represent the mean ± SEM. *n* = 3.

An essential biomarker of senescent cells is an increase in senescence‐associated beta‐galactosidase (SA β‐gal). Therefore, the effect of blue light on SA β‐gal activity was investigated. Dermal fibroblasts were irradiated with doses of blue light ranging from 5 to 50 J/cm^2^, before staining for SA β‐gal was performed. SA β‐gal increases dose‐dependently in response to blue light exposure, as displayed across all images (Figure 5A). There is a gradual increase in SA β‐gal accumulation observed in cells exposed to 10, 15, and 25 J/cm^2^, with a noticeable increase in blue staining (Figure 5A). There appears to be a plateau in SA β‐gal activity in doses above 25 J/cm^2^, as confirmed by the images acquired and quantitative analysis (Figure 5A + 5B). Quantification of SA β‐gal activity verified our image observations, with a dose‐dependent increase in SA β‐gal expression compared to the non‐irradiated control. This increased expression peaked in the group exposed to 25 J/cm^2^, with a 178% increase compared to the control (*p* = ≤ 0.01) (Figure 5B), before a slight but still statistically significant decrease in the 50 J/cm^2^ group (*p* = ≤ 0.05) (Figure 5B).

## 4 Discussion

The findings presented here represent novel work and suggest that blue light may have the ability to induce senescence in dermal fibroblasts. The 50 J/cm^2^ represents the equivalent of 1 h of solar exposure expected at noon in midsummer in the Mediterranean at 45° latitude, making the doses demonstrated here of particular physiological relevance [2]. Previous research has linked cellular senescence with premature aging in the skin, making our research of particular interest. Our findings show that blue light has the ability to induce immediate DNA damage by way of increased γH2AX expression. These increased double‐strand breaks can be observed at blue light doses ranging from 5 J/cm^2^ to 100 J/cm^2^, without any increase in nuclear area; this indicates that cellular hypertrophy may be delayed in onset, rather than an immediate effect. A key characteristic of senescent cell morphology is hypertrophy with upregulation of P21 [27]. Our findings support this, with statistically significant increased P21 fluorescent intensity 16 h after irradiation across all doses of blue light, ranging from 5 J/cm^2^ to 50 J/cm^2^, combined with statistically significant increased nuclear area; this once again suggests that cellular hypertrophy may be delayed in onset. The simultaneous increase in both γH2AX and P21 expression has previously been considered a hallmark of cellular senescence [28].

Our previous research displayed the ability of blue light to induce mitochondrial DNA damage and increase ROS production [12]; we now hypothesize that blue light induces both nuclear and mitochondrial DNA damage simultaneously. Aging and senescent cells have been shown to display a decreased ability to repair DNA damage [29]. This suggests that the increase in DNA damage presented in our work may lead to a state of permanent cellular senescence. Increased ROS production has already been linked to DNA damage and NFκB activation in gene promoter areas [30, 31]. Our findings support this, with increased NFκB nuclear translocation in a dose‐dependent manner and altered gene expression of various senescent markers. This altered gene expression suggests that normal skin physiology may be affected by blue light exposure.

Importantly, we demonstrate that blue light induces a significant upregulation of P21 expression. P21 is a key biomarker of early senescence [24], suggesting that blue light has the ability to trigger senescent processes in dermal fibroblasts. Gene expression analysis was conducted 16 h after blue light exposure, which could account for the lack of increased P16 expression, as it is associated with late‐stage senescence [24]. The observed lack of change in BCL2 expression, which is a pro‐survival protein associated with senescent cells, also supports this; we speculate that early‐onset senescence would be unlikely to trigger apoptotic mechanisms and therefore fail to induce an increase in BCL2 expression at this point. It would be necessary to conduct further research to confirm this. Interestingly, increased expression of P21 has also been linked to decreased apoptosis, which could also account for the lack of upregulation of the pro‐survival protein BCL2 at this stage [28].

The similar gene expression profile of P16 and P53 warrants further investigation. Both display decreased expression in response to blue light irradiation. P53 has been previously associated with the induction of apoptosis in skin [32]; our results demonstrate that P53 displays a trend of downregulation in response to blue light, which, taken together with the upregulation of P21, also supports our suggestion that the induction of apoptotic mechanisms may be delayed and account for the absent upregulation of BCL2. Future research could focus on the continuing effects of blue light longer after the initial exposure. The dose‐dependent increased expression of NFκB as seen in both qPCR and immunofluorescence suggests that blue light may have the ability to activate the SASP and bystander effects in neighboring cells, which is a key hallmark of cellular senescence [18].

The ability of blue light to induce cellular senescence is supported by its ability to induce increased SA β‐gal activity. We observed a gradual increase in SA β‐gal activity in response to blue light irradiation, with statistical significance observed in both groups exposed to 25 J/cm^2^ and 50 J/cm^2^. Beta‐galactosidase is only present in senescent cells, while being absent in tumor, immortal, and quiescent cells [33]. SA β‐gal activity is independent of DNA synthesis and is also not present in cells that have an arrested cell cycle, making it a very reliable marker of cellular senescence [17, 28].

Taken together, our findings demonstrate the ability of blue light to induce senescence in human dermal fibroblasts. This may play a key role in an aging skin phenotype, where senescent cells have previously been shown to induce extracellular matrix (ECM) remodeling [34]. Dermal fibroblasts produce both collagen and elastin, both of which are key to the maintenance of skin integrity [35]. Our research strongly suggests that blue light may induce cellular senescence, leading to extracellular matrix (ECM) remodeling, loss of skin integrity, and ultimately, premature aging of the skin.

## Author Contributions

Mark Birch‐Machin, as the senior and corresponding author, co‐designed the research presented with Helen McNish, Mruthyunjaya Swamy Mathapathi, Katarzyna Figlak, and Anita Damodaran. All experiments were performed by Helen McNish. Helen McNish wrote the paper. All authors contributed to the editing of the paper. Mruthyunjaya Swamy Mathapathi, Katarzyna Figlak, and Anita Damodaran supervised the project as representatives of Unilever PLC. The coded script used in Image J for immunofluorescence image analysis was formulated by Dr Glyn Nelson of the Newcastle University Bioimaging unit.

## Funding

The work in this study was funded by the Biological Sciences Research Council (BBSRC) in collaboration with Unilever (UKRI/BBSRC, BB/X511390/1), awarded to Mark Birch‐Machin as principal investigator.

## Conflicts of Interest

The authors declare no conflicts of interest.

## Data Availability

The data that support the findings of this study are available on request from the corresponding author. The data are not publicly available due to privacy or ethical restrictions.
